# Bioactive Keratin and Fibroin Nanoparticles: An Overview of Their Preparation Strategies

**DOI:** 10.3390/nano12091406

**Published:** 2022-04-20

**Authors:** Marta Giannelli, Andrea Guerrini, Marco Ballestri, Annalisa Aluigi, Roberto Zamboni, Giovanna Sotgiu, Tamara Posati

**Affiliations:** Consiglio Nazionale delle Ricerche, Istituto per la Sintesi Organica e la Fotoreattività (CNR-ISOF), Via Piero Gobetti 101, 40129 Bologna, Italy; andrea.guerrini@isof.cnr.it (A.G.); marco.ballestri@isof.cnr.it (M.B.); annalisa.aluigi@isof.cnr.it (A.A.); roberto.zamboni@isof.cnr.it (R.Z.); giovanna.sotgiu@isof.cnr.it (G.S.); tamara.posati@isof.cnr.it (T.P.)

**Keywords:** nanoparticles, drug delivery systems, protein-based scaffolds, targeted cancer therapy

## Abstract

In recent years, several studies have focused their attention on the preparation of biocompatible and biodegradable nanocarriers of potential interest in the biomedical field, ranging from drug delivery systems to imaging and diagnosis. In this regard, natural biomolecules—such as proteins—represent an attractive alternative to synthetic polymers or inorganic materials, thanks to their numerous advantages, such as biocompatibility, biodegradability, and low immunogenicity. Among the most interesting proteins, keratin extracted from wool and feathers, as well as fibroin extracted from *Bombyx mori* cocoons, possess all of the abovementioned features required for biomedical applications. In the present review, we therefore aim to give an overview of the most important and efficient methodologies for obtaining drug-loaded keratin and fibroin nanoparticles, and of their potential for biomedical applications.

## 1. Introduction

Since the submicron “magic bullet” therapy used to target drugs to specific sites was developed by Paul Ehrlich (Nobel Prize for Medicine in 1908) [1], science has made unbelievable progress in the drug delivery field. In the past few years, the development of nanotechnology has led to several advantages in many disciplines—especially in nanomedicine, wherein nanoscale materials are employed as imaging agents or for drug delivery applications [2]. Furthermore, researchers have focused their studies on smart carriers capable of selectively transporting the drug to the target tissue in order to improve therapeutic effectiveness while reducing unwanted effects that are often caused by the wide distribution of pharmaceutical molecules in the human body [3,4,5,6,7].

Among the several types of these nanoscale materials, nanoparticles have proven to be a very encouraging biomedical platform, due to their small diameters, good ability to penetrate into cells, and large surface area [8,9]. As a matter of fact, nanoparticles can (1) increase the bioavailability of medicines by reducing their degradation rate, (2) perform protective activity against drug degradation, (3) improve the solubility of hydrophobic drugs, (4) control drug release, (5) modify pharmacokinetics, (6) selectively increase cellular uptake, and (7) target disease sites specifically [10,11].

Nanoparticles can be divided into three categories: (1) inorganic, (2) lipid-based, and (3) polymer-based, the latter of which are the most interesting in the biomedical field, due to their versatility [11]. More specifically, synthetic polymers are more stable, and allow more reproducible nanoparticles to be obtained. However, they are often bio-incompatible; therefore, historically, the FDA has rarely approved polymeric nanomaterials from synthetic polymers [12]. In order to overcome this issue, researchers have focused their attention on natural polymers—such as proteins—as they show renewability, biocompatibility, biodegradability, non-immunogenicity, and non-toxicity [13], allowing them to be more easily approved by regulatory entities. Another benefit of proteins is the possibility to use several methodologies to load drugs, thanks to their defined primary structure [14]. One of the most common proteins used in nanomedicine is human serum albumin (HSA); in fact, nanocarriers based on albumin are biodegradable, easy to prepare, and can accommodate several types of drugs, due to the presence of reactive functional groups in its structure [15]. In this regard, the FDA approved the use of albumin-bound paclitaxel nanoparticles (Abraxane) for treating some types of tumours, such as metastatic breast cancer, lung cancer, and advanced pancreatic cancer [16].

Among the several biopolymers used for nanocarrier fabrication, keratin and silk fibroin have been shown to be excellent candidates for the production of nanoparticles for drug delivery applications.

Keratin is the most abundant biodegradable, non-food protein, being the principal constituent of wool, nails, hairs, horns, and feathers [17]. The structure of this polymer is rich in cysteines, which give mechanical, thermal, and chemical stability; indeed, keratin presents good stability in water, which is a great advantage for biomedical applications [18]. Moreover, in its primary structure there are two motifs—“Arg-Gly-Asp” (RGD) and “Leu-Asp-Val” (LDV)—which can create bonds with ligands of the cell surface and promote cell adhesion [19]. Fibroin is a natural polymeric biomaterial that possesses interesting features for biomedical applications, such as excellent structural properties, mechanical strength, controlled biodegradability, and non-inflammatory activity [20,21,22]. This protein can be extracted from the fibres produced by silkworms (*Bombyx mori*) and spiders, such as *Araneus diadematus* and *Nephila clavipes* [23,24]. The FDA has approved its application for sutures, tissue regeneration, coating devices, and drug delivery systems [25,26]. Interestingly, both of these polymers are characterised by high molecular weights (45–60 kDa for keratin extracted from wool, and around 350 kDa for fibroin from *Bombyx mori* cocoons) that make these proteins particularly suitable for processing into several types of structural materials, such as films, sponges, electrospun fibres, and nanoparticles [27,28,29,30,31]. All of these formats have recently found potential interest in several fields, ranging from optoelectronic to biomedical applications. As regards the use of keratin as nanoparticles, some studies have demonstrated the efficacy of keratin-based nanoparticles in anticancer drug delivery systems, thanks to their ability to reach the tumour site and to gradually release the drug [18,32]. Another advantage of keratin is that in physiological conditions it is negatively charged; therefore, it can create interactions with positively charged molecules in order to obtain a more effective transport [33]. It is already known that fibroin-based nanoparticles are also able to deliver both hydrophilic and hydrophobic drugs, such as aspirin [34], as well as anticancer agents such as doxorubicin [35], and bioactive molecules.

For all of these reasons, in this review we aim to bring together all of the main methodologies used to prepare keratin and fibroin nanoparticles, focusing on their applications in the drug delivery and drug release fields.

## 2. Synthesis of Keratin and Silk Fibroin Nanoparticles

Polymeric nanoparticles—both natural and synthetic—have been prepared by several methods, depending on the properties required for their application and on the physicochemical characteristics of the drug [36,37]. For the synthesis of protein-based nanoparticles, there are a lot of approaches that rely on a balance between electrostatic and repulsive forces in the protein chains [38]. In addition, throughout the formation of nanoparticles, conformational variations occur due to concentration, pH value, ionic force, and the solvent used. In this review, the main techniques employed to prepare keratin and fibroin nanoparticles are reported.

### 2.1. Desolvation

**Figure 1:**
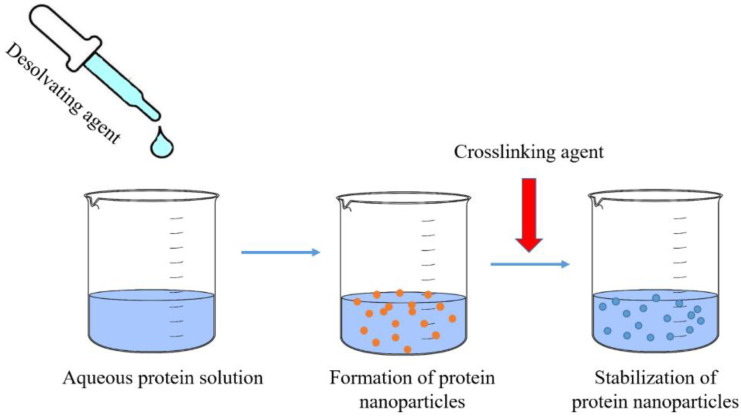
Preparation of nanoparticles via the desolvation method.

Desolvation is the most frequently used technique to formulate protein-based nanoparticles, due to simplicity of operations, mild conditions, and the ability to obtain small particle sizes [39]. Figure 1 shows a schematic representation of the desolvation method.

In brief, the dropwise addition of a desolvation agent to an aqueous solution of protein reduces the availability of water molecules and causes dehydration of polymer chains. When the hydration becomes too low, chains aggregate, and the protein precipitates in the form of nanoparticles. Sometimes, nanoparticles are not stable; therefore, crosslinking agents must be added [15,40].

**Figure 2:**
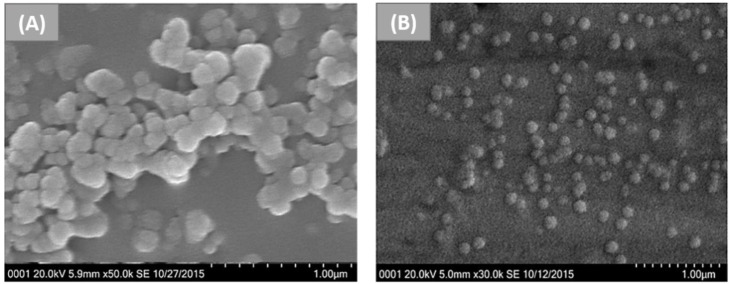
SEM images of KDNPs with different magnifications: (**A**) x 50 K and (**B**) x 30 K. Reprinted and adapted with permission from [41]. Copyright Elsevier Clearance Center 2022.

Li et al. prepared keratin nanoparticles loaded with doxorubicin (DOX) via the desolvation technique, using ethanol as a desolvation agent, followed by the electrostatic adsorption of the drug [41]. The keratin used in this work was extracted from human hair, and then nanoparticles were prepared. In brief, they added ethanol to the aqueous protein solution in order to decrease the solubility of keratin until the formation of nanoparticles. Then, they used glutaraldehyde to crosslink the nanoparticles to make them stable in solution. Finally, DOX-loaded keratin nanoparticles (KDNPs) were formed by suspending keratin nanoparticles (KNPs) in DOX solution; thus, the drug loading content (LC) and drug encapsulation efficiency were evaluated. Keratin nanoparticles were characterised in terms of size by DLS and SEM (Figure 2), showing a diameter of around 214.8 nm and 150 nm, respectively. On the other hand, KDNPs were proven to have similar diameters: 220.8 nm by DLS, and around 150 nm by SEM.

Since the purpose of their work was to prepare a drug delivery system based on protein nanoparticles, the authors also studied the release of DOX under physiological conditions (PBS pH 7.4) and in acidic environments, in order to simulate the pH of the tumour area. At first, the pharmaceutical release seemed to be quite slow at both pH levels; subsequently, the release took two different paths: at pH 7.4 only 35% of DOX was released, while at pH 5.3 around 50% was released, due to the protonation of both DOX and keratin. The authors also studied the drug release profile depending on glutathione (GSH) concentration, showing that more than 60% of DOX was released at the GSH level of the tumour environment, while in the absence of GSH the release was only about 18%. Finally, the MTT assay led Li et al. to demonstrate the non-toxicity of KNPs and the high antitumour efficiency of keratin nanoparticles charged with doxorubicin against lung cancer cells.

Photodynamic therapy (PDT) is a non-surgical clinical treatment that can be used for infections and malignant cancers [42,43]. This technique exploits the activity of photosensitizers (PSs), which can induce ROS formation upon irradiation with appropriate wavelengths of light [44]. Reactive oxygen species cause cells’ proliferation to decline, and sometimes cause the death of cells [45,46]. The penetration of the light in the tissue was studied by Ash et al. [47], and it was reported that red light penetrates at 4–5 mm. On the other hand, PSs have several side effects, i.e., lack of specificity and high tendency to aggregate in aqueous media [48]; thus, in recent years, researchers have focused their attention on the development of loaded nanoparticles, which can act as delivery systems, transporting the drug to the desired site and promoting its absorption [49]. Several studies have reported that the penetration of the light is also enough to activate nanoparticles—i.e., keratin-based ones—loaded with different photosensitizers into the tumour within the bone tissue [50,51].

**Figure 3:**
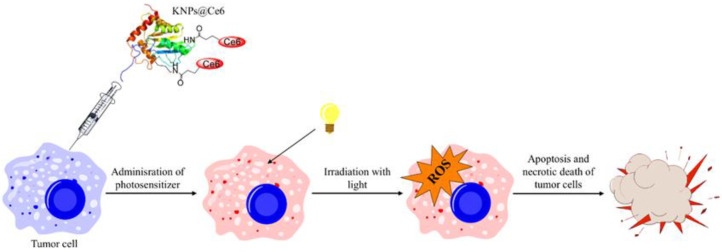
Schematisation of PDT therapy with KNPs@Ce6.

In this context, for the first time, Aluigi et al. described the preparation via desolvation of keratin nanoparticles conjugated with chlorin e6 (Ce6)—a second-generation photosensitizer [14]. In this regard, keratin was extracted from Merino wool as previously described [52], and Ce6 was covalently bound to the keratin amino groups via EDC/NHS coupling in DMSO as a solvent. The protein conjugated with Ce6 was purified by dialysis against NaHCO_3_ buffer for 2 days, and finally freeze-dried to obtain a powder. In order to prepare Ker–Ce6 nanoparticles (KNPs@Ce6), ethanol was used as a desolvation agent, because it is able to decrease the protein solubility. Firstly, the powder was solubilised in NaHCO_3_ buffer, and then ethanol was added under constant stirring at room temperature to precipitate nanoparticles. Therefore, glutaraldehyde (8%) was added to stabilise KNPs@Ce6, and the solution was purified by three cycles of centrifugation in water. This procedure allowed stable nanoparticles with hydrodynamic diameters around 147 nm and a Ce6 loading ratio of about 45% to be obtained, which were evaluated through UV–Vis measurements on nanoparticle suspensions. Further characterisations showed that KNPs@Ce6 displayed an excellent ability to cross the tumour cell membranes in both osteosarcoma and glioblastoma, and to produce a large amount of reactive oxygen species (ROS) upon light irradiation at the tumour site [53], as schematically shown in Figure 3.

**Figure 4:**
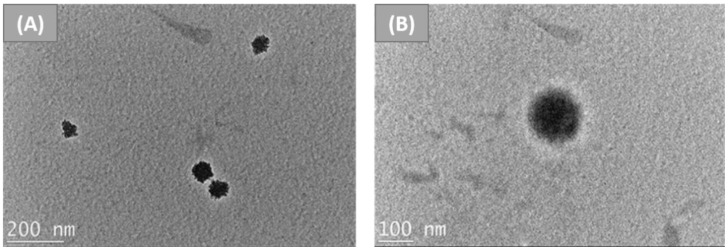
TEM images of (**A**) SF nanoparticles prepared from *Bombyx mori*, and (**B**) individual SF nanoparticles. Reprinted and adapted with permission from [54]. Copyright Elsevier Clearance Center 2022.

Similarly, Kundu et al. reported the preparation of silk-fibroin-based nanoparticles via the desolvation method [54]. In brief, silk fibroin (SF) aqueous solution was obtained by extraction from *Bombyx mori* cocoons using a standard procedure [55]. SF solution was added dropwise to DMSO solution under constant low magnetic stirring; the formation of nanoparticles was confirmed by the precipitate produced at the bottom. Then, nanoparticles were separated and purified by centrifugation with deionised water. Hence, they were redispersed in deionised water by sonication, and finally filtered using a 0.45 µm syringe filter to remove dust particles or contaminants. Finally, fluorescein isothiocyanate was linked to SF nanoparticles. The nanoparticles obtained had negative charges on the surface, as demonstrated by the reported value of zeta potential (−24.41 mV), which prevented further agglomeration of the particles; therefore, they were stable in deionised water and in cell culture media containing serum over a long period of time. FTIR spectra demonstrated that, during the formation of nanoparticles, the conformation of silk went from I to II, explaining the nanoparticles’ insolubility. A light-scattering particle analyser demonstrated that the nanoparticles’ hydrodynamic diameter was around 177 nm, and TEM analysis showed that the particles were spherical granules without signs of adhesion (Figure 4). Cytotoxicity and cell cycle assays on murine fibroblast cells were also carried out, and demonstrated that silk fibroin nanoparticles were moderately non-toxic to the cells. Overall, this study shows a smart methodology to fabricate silk fibroin nanoparticles, which can be used for drug delivery.

Additionally, Zhang et al. reported the preparation of silk fibroin nanoparticles starting from an aqueous solution of regenerated SF, which was mixed with water-miscible protonic organic solvents (e.g., ethanol and methanol) or with aprotic organic solvents (e.g., acetone) [56]. The formation of nanoparticles depends on the configuration transition from random coils and α-helices to β-sheets. Briefly, the regenerated SF solution, obtained by extraction from *Bombyx mori* cocoons, was quickly added to at least 70% (*v*/*v*) of the final mixture volume of water-miscible organic solvent—specifically, acetone. This process caused an increase in the number of β-sheet structures; therefore, the protein started to precipitate, and nanoparticles were formed. Then, the particles were purified by centrifugation, redispersed in deionised water, and finally lyophilised. TEM and SEM analysis showed that silk fibroin nanoparticles were globular granules with some microparticles aggregating together, and they were in the range of 35–125 nm in diameter. Finally, the authors demonstrated that the presence of SF nanoparticles did not affect the growth and propagation of bacteria, whether Gram-positive or Gram-negative.

### 2.2. Electrospraying

Electrospraying is a suitable technique to prepare micro- and nanoparticles using various materials, such as natural biopolymers. This process is based on the use of an electric field, which is applied to a drop, generating an electric charge, called Coulomb force. When this force becomes greater than the cohesive force, a diminution in the surface tension occurs, and nanoparticles are obtained [57,58]. Over time, variations on the conventional electrospraying technique have been developed, such as:

- Electrospraying in solution: the electrosprayed polymer solution is dropped into an immiscible solution, which contains a crosslinking agent, in order to obtain stable particles [59];
- Coaxial electrospraying: this method allows a core–shell structure to be obtained, starting from two different polymer solutions, which are loaded into separate syringes, and one needle is put inside the other [60,61];
- Electrospraying by deposition on a substrate: the polymer solution is electrosprayed on a solid substrate in order to cover its surface; this method is mainly used for application in solar cells [62,63,64].

Moreover, electrospraying is widely used due to its facility, simple control of parameters, and the possibility to obtain products in one step. In the literature, numerous studies have reported the synthesis of protein nanoparticles via electrospraying methods in order to develop alternative drug delivery systems, since this technique allows drugs to be encapsulated into protein nanoparticles [65,66,67].

In this regard, in 2014, Ebrahimgol et al. documented the preparation of keratin nanoparticles by electrospraying, starting from Merino wool [68]. In particular, keratin was extracted from wool using reduction hydrolysis [69], which enables a powder of protein to be obtained as a result of the freeze-drying process. Keratin was then solubilised in formic acid at 70 °C for 24 h under magnetic stirring, using a concentration about 0.3% *w/v*. The solution was placed into a syringe with a needle, and then connected to a positive electrode, while the aluminium foil collector was connected to the negative electrode and, finally, high voltage was applied. The authors also studied several concentrations of keratin solution, concluding that when the concentration was higher than 0.5%, the solution could not be electrosprayed. SEM images showed that the nanoparticles were spherical and their diameter was around 36–72 nm, depending on the electrospraying conditions. Then, Ebrahimgol et al. evaluated the effect of nozzle–collector distance for two feed rate levels (0.02 mL/h and 0.04 mL/h). Specifically, it was discovered that the dimensions of the particles decreased as the nozzle–collector distance increased and as the feed rate decreased, while they were not influenced by the applied voltage. Collectively, this paper showed a new smart methodology to prepare keratin nanoparticles in a controlled manner.

Guo et al., in 2018 [70], used an electrospraying technique to modify polymeric nanofibers’ surface with keratin nanoparticles (KNPs), in order to improve the hydrophilicity and biocompatibility of polyvinyl alcohol (PVA) nanofibers [71]. In the literature, some studies have reported the preparation of keratin–PVA blended nanofibers, but the use of protein caused a lot of problems due to its low viscosity and poor spinning properties [72,73,74]; therefore, the purpose of this work was to offer a strategy to overcome these drawbacks. Briefly, oxidative keratin (keratose, KOS) was extracted from human hair via an oxidation method [72], and PVA nanofibers were electrospun using a 10% (*w/v*) solution in ethanol. Then, the KNPs were directly sprayed onto the PVA nanofibers by electrospray deposition, using different amounts of keratose (1.0, 1.5, 2.0, and 2.5%). The process was performed on aluminium foil paper, and the nanofibers were dried at 50 °C for 24 h. SEM images demonstrated that the KOS nanoparticles’ diameters were approximately 250–350 nm, and that they adhered to the PVA nanofibers. Additionally, KNP–PVA nanofibers showed improved performance in neural cell morphology, adhesion, and proliferation, depending on the presence of KNPs, compared to pure PVA nanofibers. Therefore, Guo et al. demonstrated the possibility to functionalise polymeric nanofibers with KNPs by using electrospray deposition in order to improve the biocompatibility and mechanical properties for potential neural tissue applications.

**Figure 5:**
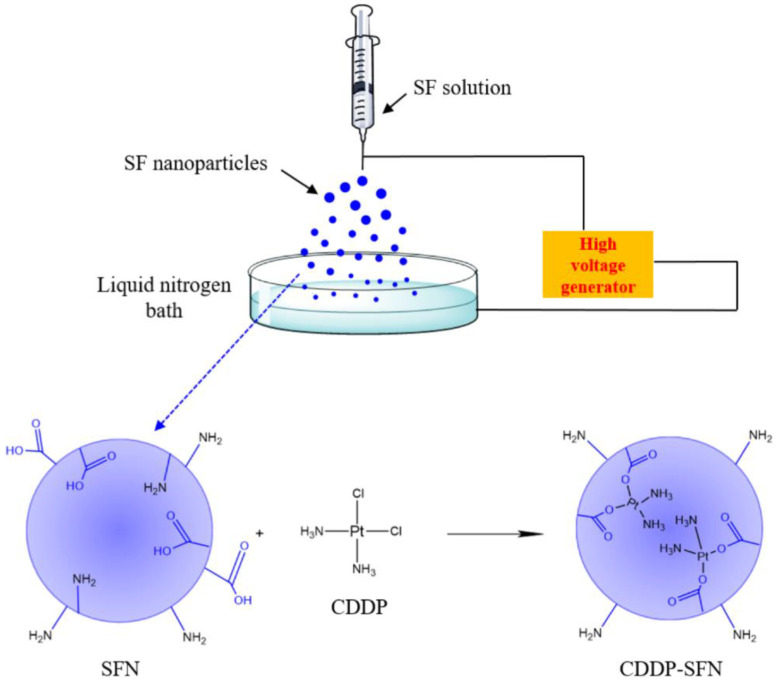
Preparation of SF nanoparticles loaded with CDDP via electrospraying technique.

In 2014, Qu et al. [75] demonstrated the preparation of silk fibroin nanoparticles (SFN) by electrospraying to produce controlled-release carriers of cisplatin (CDDP)—a chemotherapeutic agent—in order to reduce its cytotoxicity and adverse effects on healthy tissue. Silk fibroin (SF) was extracted from raw silk fibres as previously described by Wang et al. [76], obtaining an aqueous protein solution. Then, glycerin was added to the SF solution (30% vs. protein). SFNs were prepared by electrospraying, using different electrostatic voltages (between 13 and 16 kV), and the droplets were continuously collected and frozen in a liquid nitrogen bath. SFNs were freeze-dried, redispersed in deionised water, and centrifuged in order to remove impurities. CDDP was then incorporated into SF nanoparticles via a ligand-exchange reaction of Pt from the chloride to the carboxyl group in the SF structure, as shown in Figure 5.

Briefly, an amount of cisplatin powder (about 20% *w/w*) was added to an aqueous nanoparticle solution, and then the system was stirred and, finally, dialysed. The encapsulation efficiency and the drug loading content were about 87.4% and 11.4%, respectively. SEM images allowed the researchers to demonstrate that the SFNs obtained were all spherical and well dispersed, while their diameters varied with the voltage. In particular, nanoparticles prepared under 14 kV and 15 kV showed smaller diameters than those obtained under 13 and 16 kV, probably due to the instability of the jet flow. Then, in vitro release of CDDP-loaded nanoparticles demonstrated that the cisplatin was released slowly, allowing a decrease in unwanted effects on healthy tissues. In vitro cellular cytotoxicity tests on murine fibroblast cells confirmed that CDDP–SFN had no apparent toxic effect on normal cells, while free CDDP displayed strong toxicity to them. Finally, these nanoparticles demonstrated a noteworthy inhibitory effect on lung cancer cells, which they could penetrate via adsorption endocytosis.

Similarly, Cao et al. [77], in 2017, proposed the coaxial electrospraying technique to produce a novel potential drug delivery system for doxorubicin (DOX), based on core–shell PVA–silk fibroin nanoparticles. This method used two distinct capillaries: the PVA ethanol solution, containing DOX, was injected into the inner channel, while the SF solution, extracted from *Bombyx mori* cocoons and then dissolved in HFIP, was injected into the outer channel. The authors studied the effect of PVA content on nanoparticle dimensions, concluding that the size diameters were controlled by polymer concentrations in solution. In particular, particles became larger as the concentration of the polymer increased. Moreover, DOX–PVA/SF NPs were negatively charged on the surface; hence, they were stable in solution. TEM images showed the core–shell structure of particles with an inner core of DOX–PVA covered by a layer of SF. Cao et al. also studied the DOX release profile, showing that the inner PVA core was dissolved into the aqueous medium, initially facilitating the drug release. After the initial burst release, it seemed that several DOX molecules remained entrapped by SF; therefore, the release was facilitated by the use of ultrasound stimuli. Finally, the authors evaluated the cytotoxicity of the nanoparticles, finding that PVA/SF NPs were safe for human breast cancer cells, while DOX–PVA/SF NPs had high cytotoxicity to tumour cells when doxorubicin was released.

### 2.3. Self-Assembly and/or Aggregation

Self-assembly, also sometimes called aggregation when mediated by drugs, is a suitable technique used in the field of nanotechnology, because it allows stable nanometre products to be obtained under mild conditions, and without involving organic solvents or high temperatures. This process occurs when a disordered system spontaneously forms a well-organised structure as a result of specific local interactions between species [78,79,80]. The main forces involved in this process are van der Waals, hydrophobic, electrostatic, and hydrogen bonding forces, which are generally weak, but when combined together allow stable self-assembled structures to be obtained [81]. The self-assembly technique can be classified as follows [78]:

- **Static**: occurs in the absence of external factors, and is determined by the minimisation of energy;
- **Dynamic**: the process is influenced by external factors.

**Figure 6:**
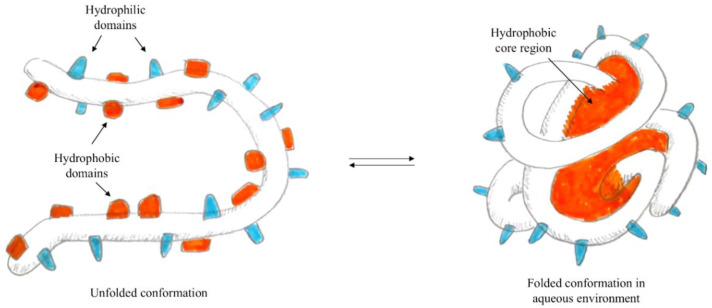
Schematisation of protein self-assembly.

In particular, when the self-assembly technique is used for the preparation of polymeric nanoparticles—i.e., proteins [82]—the presence of both hydrophilic and hydrophobic domains is exploited. In fact, in water, the proteins form micelles, where hydrophobic domains face the core while hydrophilic domains orient on the surface, as represented in Figure 6. Using the formation of interactions between hydrophobic residues of the protein and lipophilic molecules, it is possible to load the hydrophobic core of the nanoparticle with active lipophilic principles, which in their free form are insoluble in an aqueous environment.

In this regard, Liu et al. [83] reported the preparation of human hair keratin (HHK) nanoparticles loaded with mupirocin (MPC)—a novel antibacterial agent with a mode of action different from other antibiotic agents—allowing them to obtain a nano drug delivery system. Briefly, keratin was extracted from human hair by slightly modifying the procedures reported in [84], and a protein aqueous solution (10 wt%) was obtained. Meanwhile, different amounts of MPC were dissolved in PBS buffer solution. Then, the nanoparticles were formed by mixing HHK solution and MCP solution under stirring at pH 6.5. In this work, the concentrations of both keratin and MCP solutions were studied, and the researchers noted that the formation of nanoparticles did not occur when the concentration of HHK or MCP was higher than 0.5%. The dynamic light-scattering particle size analyser showed that nanoparticle diameters were about 75 nm, while SEM images confirmed that the keratin nanoparticles loaded with mupirocin had an irregular shape. In conclusion, Liu et al. demonstrated the preparation of keratin nanoparticles using self-assembly and electrostatic interaction, in order to suggest a novel drug delivery system based on a natural biopolymer.

Recently, in 2021, Du et al. [85] investigated the preparation of keratin–tannic acid complex nanoparticles, to produce a pH/GSH dual-responsive drug carrier for doxorubicin. In particular, tannic acid (TA) is usually employed as a non-toxic crosslinker for proteins [86,87], and in this work it formed complex nanoparticles together with keratin via a self-assembly process with non-covalent interactions, including hydrogen bonding and hydrophobic interaction [88]. Firstly, keratin was extracted from human hair using the reduction method described in [89]. Then, tannic acid powder was added to the aqueous keratin solution, and the system was stirred for 12 h at room temperature. The mixture was then dialysed and lyophilised in order to obtain keratin–TA nanoparticles (KNPs). Finally, the drug-loaded keratin nanoparticles (DKNPs) were prepared by exploiting the hydrophobic interactions and hydrogen bonds between KNPs and doxorubicin. TEM images showed that the nanoparticles were round/oval and well dispersed. Additionally, further characterisation showed that the diameters were about 240 nm and the zeta potential was −0.21 mV. Moreover, the authors studied the drug release profile, demonstrating that DKNPs released more doxorubicin at pH 5.0 than at pH 7.4, due to the protonation of keratin, and also that the release of the drug was facilitated by high levels of GSH, which characterise the tumour area. The MTT assay, conducted on murine fibroblast cells, demonstrated that the toxicity of the free drug was higher than the cytotoxicity of DKNPs. Furthermore, the in vitro cytotoxicity test on lung carcinoma cells confirmed the antitumour activity of DKNPs, although their inhibition was weaker than that of free DOX, due to the different mechanism of interaction with the cells. Specifically, the free drug penetrated into the nucleus via diffusion, while DKNPs were internalised by endocytosis, and then released DOX.

**Figure 7:**
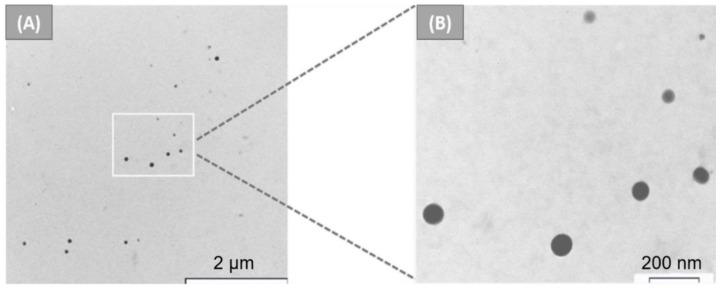
TEM images of KER–NPs–PTX at different magnifications: (**A**) scale bar 2 µm, and (**B**) scale bar 200 nm [90]. Originally published by and used with permission from Dove Medical Press Ltd.

For the first time, in 2018, Foglietta et al. [90,91] reported the application of keratin nanoparticles loaded with paclitaxel (PTX) as a novel drug delivery system (KER–NPs–PTX) to overcome the issues related to the use of taxanes, i.e., toxicity and poor water solubility [92]. Briefly, keratin powder, obtained from wool as previously reported [14], was dissolved in PBS solution, and then different amounts of PTX in ethanol were added under vigorous stirring. Characterisations of KER–NPs–PTX showed that the nanoparticles’ dimensions increased with PXT loading, ranging from 5 to 23% (wt). Additionally, TEM analysis (Figure 7) showed that the nanoparticles were spherical, with a smooth surface morphology.

Stability studies in PBS and FBS/H_2_O indicated the high stability of nanoparticles, while cytotoxicity tests on human breast cancer cells demonstrated that KNPs were safe for cell lines. Finally, the authors studied KER–NPs–PTX’s activity against some different breast cancer models, showing the ability to inhibit tumour cell viability and to induce apoptosis. In summary, Foglietta et al. demonstrated the capacity of keratin to incorporate lipophilic drugs with high loading ratios, and the possibility of using KER–NPs as a novel drug delivery system.

Following the works reported above, Busi et al. [93] studied the preparation via drug-mediated aggregation of keratin nanoparticles loaded with the R enantiomer of 9-hydroxystearic acid (9R), which exerted an in vitro growth-inhibitory effect on human colon carcinoma cells. In brief, the authors synthesised 9R as described in [94], and then they added an ethanol solution of 9R to the aqueous solution of keratin under vigorous stirring, to obtain 9R-loaded keratin nanoparticles (9R@ker NPs). The 9R@ker NPs were characterised, demonstrating them to have an average diameter of around 160 nm and to be negatively charged in water, due to the SO_3_^−^ and COO^−^ groups of keratin. Moreover, the effect on human colon carcinoma cell proliferation was studied, concluding that 9R@ker NPs caused a decrease in cell proliferation comparable to the activity of the free drug.

**Figure 8:**
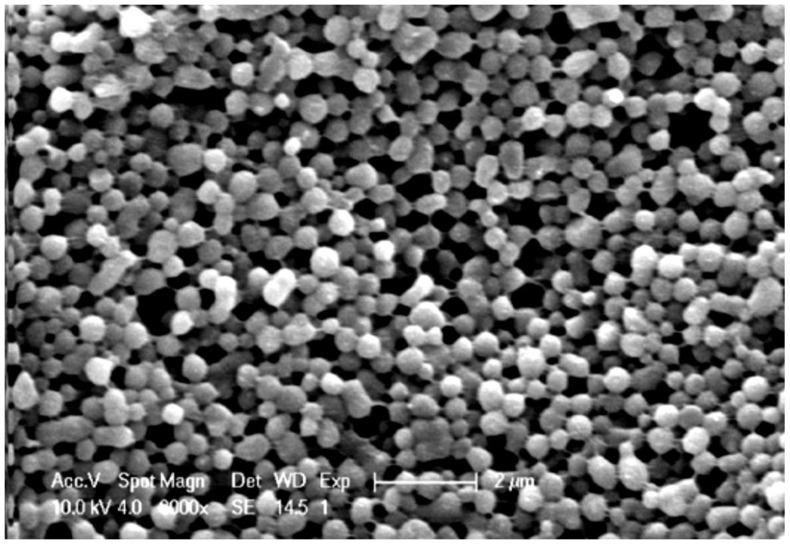
SEM image of silk fibroin nanoparticles. Reprinted and adapted with permission from [95]. Copyright Elsevier Clearance Center 2022.

On the other hand, in 2011, Shi et al. [95] demonstrated the possibility to also use silk fibroin nanoparticles as a drug delivery system, by preparing silk fibroin (SF) nanoparticles via a self-assembly technique, and studying the loading and subsequent release of model drugs (i.e., RhB, RITC–dextran, FITC–BSA). Briefly, aqueous SF solution was obtained from *Bombyx mori* cocoons [96] and mixed with specific amounts of drugs. Then, the solution was added to ethanol and vortexed. PVA solution was then added to the silk–ethanol mixture, in order to prevent the agglomeration of particles. Finally, the solution was placed in a freezer for 24 h, and the nanoparticles were obtained after centrifugation. SEM images (Figure 8) showed that the average diameter was about 980 nm, and that the nanoparticles were round but partially aggregated together.

The loading studies confirmed that the encapsulation efficiency of hydrophilic drugs, such as RITC–dextran, increased with increased loading, in contrast to the behaviour of hydrophobic drugs (i.e., FITC–BSA and RhB). Release studies showed that RhB was released more slowly than the other two drugs, probably due to its positive charge, which could attach to proteins via electrostatic and hydrophobic interactions. Finally, the alamarBlue assay demonstrated that osteoblasts cultured with drug-loaded SF nanoparticles showed higher cell viability compared to the control group.

Additionally, Li et al. [97], in 2016, investigated the anticancer efficacy in the treatment of breast carcinoma of 5-fluorouracil (5-FU)—a highly efficient chemotherapeutic agent—and curcumin—a promising candidate in clinical trials for cancer treatment—loaded with silk fibroin (SF) nanoparticles obtained via a self-assembly technique. Briefly, the aqueous silk solution was obtained from *Bombyx mori* cocoons as previously described [98], while curcumin and 5-FU were solubilised in alcohol. Then, the solution of 5-FU was added dropwise to the SF solution, followed by the dropwise addition of the curcumin solution. The mixture was stirred for 1 day, and then it was centrifuged and dialysed in order to purify the nanoparticles. TEM analysis showed that the nanoparticle diameters were about 50–250 nm, while the UV–Vis spectrum revealed the characteristic peaks of 5-FU (265 nm) and curcumin (425 nm), demonstrating that they were successfully loaded into the fibroin nanoparticles. The experiments carried out on murine breast cancer cells showed that drug-loaded nanoparticles exhibited a chemotherapeutic effect, reducing the size of the tumours. Additionally, the in vivo tests on mice also confirmed the effectiveness against breast cancer.

Alongside these common synthesis methods used for both keratin and silk fibroins, there are others technique that are mainly used for one or both proteins.

### 2.4. Microemulsion

The term “microemulsion” indicates a thermodynamically stable dispersion of two immiscible liquids with the aid of surfactant agents [99]. A water-in-oil (*w*/*o*) microemulsion is formed when nanometre-scale droplets of water are dispersed in a hydrocarbon-based continuous phase. To stabilise the system, the surfactant generates aggregates known as reverse or inverted micelles [100]. This method is useful to produce protein nanoparticles, because it allows good control of particle sizes [39], but on the other hand it requires the use of organic solvents and surfactant agents, which must be removed at the end of the process. In the microemulsion method, the nanoparticles are formed at the *w*/*o* interface, and their sizes are regulated by protein concentration and emulsification efficiency [15]. In general, the size of the nanoparticles increases depending on the protein concentration and the volumetric ratio between the aqueous and oily phases.

**Figure 9:**
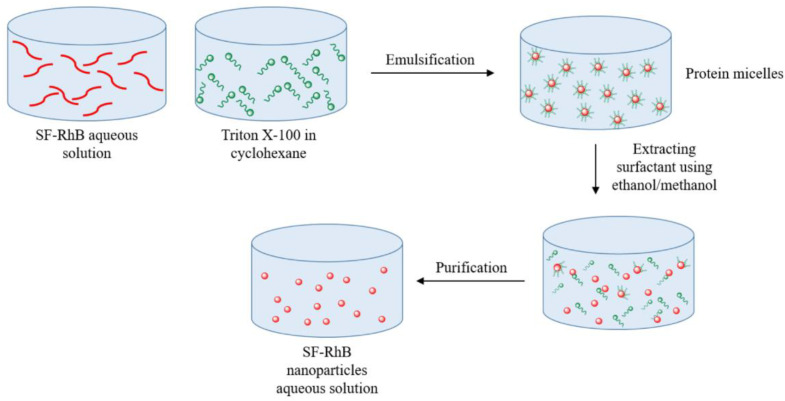
Schematic representation of the *w*/*o* microemulsion technique.

In 2008, Myung et al. [101] proposed the preparation of silk fibroin nanoparticles via a *w*/*o* microemulsion technique, to produce colour-dye-doped SF nanoparticles. In brief, an aqueous SF solution was obtained from *Bombyx mori* cocoons as previously described [102], and it was mixed with the colour dye solution (rhodamine B). As shown in Figure 9, Triton X-100 (surfactant agent) and cyclohexane (organic solvent) were added to the SF dye solution and stirred. A mixture of methanol and ethanol was then added to break the micelles and recover the nanoparticles, which were purified by dialysis. In this case, methanol has a dual function: it first breaks the microemulsion, and then stabilises the nanoparticles, inducing the β-sheet structure via the dehydration of the silk.

FTIR analysis demonstrated the presence of crystalline structures responsible for nanoparticle stability [103]. TEM images showed that the colour-dye-doped SF nanoparticles were rough and spherical, with a diameter of 167 nm. Moreover, release studies revealed that less than 7% of the rhodamine B was released; thus, the particles were stable. This paper shows a new methodology to produce silk fibroin nanoparticles loaded with a dye, which could be used in molecular imaging as a biosensor system.

### 2.5. Salting Out

Salting out is another technique to fabricate nanoparticles, and possesses several advantages, such as low cost, straightforwardness, and easy-to-maintain protein activities, avoiding the use of toxic solvents [6]. In aqueous solutions, the hydrophilic parts of the proteins interact with water molecules, forming hydrogen bonds. When the concentration of salt increases, salt ions tend to attract water molecules; therefore, protein–protein interactions take place, causing aggregation and precipitation of the proteins. The most commonly used salting-out agent is potassium phosphate, and this technique can be exploited for the preparation of novel drug nanocarriers.

In recent years, targeted drug delivery systems using magnetic carriers and an external magnetic field focused on the tumour have emerged as an encouraging approach to enhance drug accumulation at the tumour sites [104]. Among its several advantages, this technique does not chemically change the targeting ligands on the nanocarrier surface, and it can be used for a wide variety of solid tumours, because the magnetic targeting does not depend on receptors expressed on the tumour cells [105,106].

**Figure 10:**
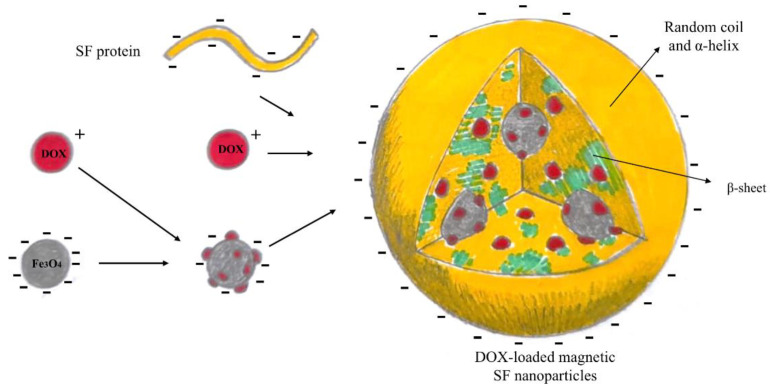
Schematic representation of DOX-loaded magnetic SF nanoparticles.

Following this path, in 2014, Tian et al. [107] developed doxorubicin-loaded magnetic silk fibroin nanoparticles using the salting-out method previously described by Kaplan et al. [108]. In brief, doxorubicin (DOX) and hydrophilic magnetic Fe_3_O_4_ nanoparticles (MNPs) were dispersed in potassium phosphate solution. Then, the aqueous silk fibroin (SF) solution, extracted from cocoons of *Bombyx mori*, was added, and the process was conducted at a very low temperature in order to obtain DOX-loaded magnetic SF nanoparticles (DMSs), as illustrated in Figure 10.

In this case, MNPs play a fundamental role—in fact, they give magnetic properties to SF nanoparticles, but also regulate the nanosystem formation, helping to induce SF β-sheet conformation [109], and the drug encapsulation, absorbing part of the DOX on their surface. The drug release profile showed that the low-pH environment accelerated the DOX release, probably due to the electrostatic interaction between the SFs and the drug at acidic pH. After obtaining good results in the internalisation and intracellular drug release behaviours on human breast adenocarcinoma cells, the authors studied the magnetic tumour-targeting performances, concluding that only DMSs accumulated at the magnet-attached tumour.

In this regard, Song et al. [110] described the preparation of magnetic silk fibroin nanoparticles loaded with curcumin (CUR), for sustained drug release into breast cancer cells. Briefly, CUR was dissolved in DMSO and added to sodium phosphate solution with magnetic nanoparticles. The mixture was added to SF solution (from *Bombyx mori* cocoons) and stored for 2 h at −20 °C, in order to obtain CUR-loaded magnetic SF nanoparticles. The authors also studied the influence of salt solution on the particle formation; in particular, they were able to demonstrate that sodium phosphate allows much smaller (about 380 nm) and smoother nanoparticles to be obtained, while when using potassium phosphate they appear larger (around 1800 nm) and rougher. The reason for this behaviour is still subject to study, but researchers think that it is due to the different sizes of the ions. In fact, the sodium cation is smaller and has a higher charge density than the phosphate cation. Therefore, sodium ions are able to reduce the activation energy of the process further, producing smaller nanoparticles. The release studies showed that curcumin was released progressively without burst release; hence, the drug was homogeneously dispersed in the nanoparticles, and the process was sustained by SF degradation [111]. Finally, the authors studied the in vitro cytotoxicity in a human Caucasian breast adenocarcinoma cell line (MDA-MB-231cells), demonstrating the enhanced cytotoxicity and higher cellular uptake of CUR-loaded magnetic SF nanoparticles. In this paper, Song et al. showed a new methodology to transport hydrophobic drugs such as curcumin—which has low solubility—to the tumour area in order to provide a new drug delivery system.

### 2.6. Ionic Gelation

Ionic gelation is commonly used for nanoparticle preparation, because it is non-toxic, organic-solvent-free, suitable, and controllable [112]. Moreover, this technique can avoid the use of crosslinkers and surfactant agents, which may cause the denaturation of proteins [113]. Ionic gelation is based on the electrostatic interactions between the negatively charged groups and the positively charged ones, which tend to form nanoparticles.

In the literature, several studies have reported the preparation of chitosan nanoparticles via ionic gelation [114,115] for biomedical applications. Recently, researchers have also focused their attention on the preparation of keratin nanosystems using this technique. In particular, in the case of keratin, the large amount of carboxylate groups (-COO) can be exploited to create strong bonds with cationic drugs.

In 2015, Zhi et al. [18] studied the drug-induced ionic gelation process in order to obtain keratin nanoparticles loaded with chlorohexidine (CHX) (KCNPs)—a drug used to treat and prevent skin and mucosal infections. Aqueous keratin solution was extracted from human hair, following the procedure reported in [116], and the aqueous CHX solution was added dropwise in order to facilitate the electrostatic interactions between the carboxylate groups of keratin and the positively charged drug molecules. TEM images showed that the KCNPs were solid spheres with a diameter of around 176 nm, while the zeta potential (−39.1 mV) measurements demonstrated that the nanoparticles had a negative surface charge and, thus, were stable in aqueous solution. Zhi et al. studied the cytotoxicity towards fibroblast cells via MTT assay, showing that KCNPs were low-dose toxic and that they maintained their antibacterial activity against *E. coli* and *S. aureus*. An ideal antitumour delivery system can regulate the release as a function of pH conditions in order to promote the drug release—especially in the tumour tissues, due to their acidic environment [117]. Therefore, the in vitro release tests demonstrated that the drug release could be regulated by pH conditions—in particular, CHX showed a faster and long-term release in an acidic environment.

**Figure 11:**
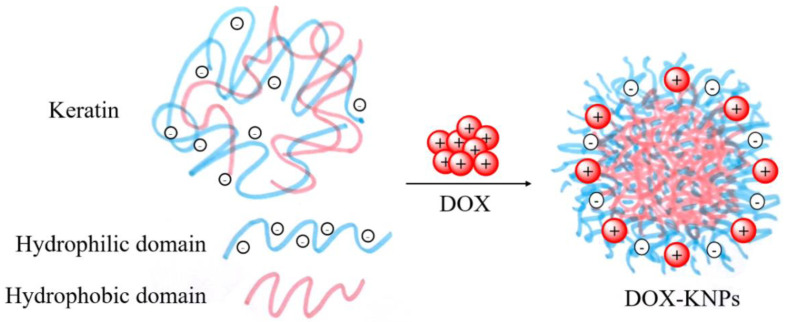
Schematic representation of DOX-loaded keratin nanoparticles.

Similarly, Aluigi et al. [118] studied the production of keratin nanoparticles via ionic gelation, obtaining a stable system that could be loaded with doxorubicin (DOX) in order to reduce the cardiotoxicity of the drug. Operationally, different amounts of DOX solution were added dropwise to the aqueous solution of keratin, extracted from wool by sulphitolysis, and the mixture was maintained under stirring and stored at 4 °C overnight. After that, it was dialysed and centrifuged to obtain purified nanoparticles (DOX–KNPs), as represented in Figure 11.

The whole process was conducted in water: in fact, at this pH, DOX is mostly in its cationic form, and is thus attracted by Bunte salts, developing nanoparticles via electrostatic complexation, as demonstrated by FTIR analysis monitoring the peak at 1025 cm^−1^ (related to symmetric S=O stretching of Bunte salts) [119].

The authors also studied the release of DOX, showing that the drug release reached 60% within the first 24 h in acidic conditions, while in a physiological environment it was only about 38%.

## 3. Conclusions

The aim of this paper was to give an overview of the main techniques—summarised in Table 1—used for the preparation of protein-based nanoparticles.

In particular, we focused our attention on two proteins—keratin and fibroin—which possess extraordinary properties, such as biodegradability, biocompatibility, cell adhesion capabilities, and the ability to obtain new stable systems for the controlled and modified release of drugs. These two proteins share some preparation methods—i.e., desolvation, electrospraying, and self-assembly—while some others are more specific to keratin, such as aggregation and ionic gelation, or to fibroin, i.e., microemulsion and salting out. Furthermore, all of these methodologies enable stable nanoparticles to be obtained, sometimes avoiding the use of crosslinking agents, with dimensions that can be controlled by tuning the process parameters and conditions. Several researchers have studied how to load these nanoparticles with several drugs, in order to overcome drawbacks related to their use, such as cytotoxicity and poor solubility in water. It is well known that the biodistribution of nanoparticles is regulated by the EPR (enhanced permeability and retention) effect, by which nanoparticles tend to accumulate in tumour tissue much more than in healthy tissues, releasing the drugs in a controlled manner depending on the nanoparticles’ dimensions, the hydrophobicity/hydrophilicity of the drugs, and biodegradability [120]. Moreover, proteins degrade into non-toxic byproduct amino acids that can be easily absorbed, metabolised, and excreted by the body [11]. In conclusion, the results obtained to date are very promising, and suggest the possibility to use natural biopolymers to prepare innovative drug delivery systems.

## Figures and Tables

**Table 1:** Manufacturing methods for the preparation of nanoparticles.

Methods	Advantages	Disadvantages
Desolvation	Operational simplicity; Mild conditions; Small particle size	Use of crosslinking agents and organic solvents
Electrospray	Ability to process different materials; Low cost; One-step procedure; Controllable particle size	Expensive; Complex equipment
Self-assembly/aggregation	Mild conditions; No organic solvents	Low size control
Microemulsion	Good control of particle size	Use of organic solvents and surfactant agents; Tedious purification steps
Salting out	Mild conditions; Easy to scale up; Low cost	Large particle size; Several purification steps
Ionic gelation	Organic-solvent-free; Suitable; No surfactant agents	Large particle size

## Data Availability

Not applicable.
